# Comparing the effectiveness of a hybrid and in-person courses of wheelchair service provision knowledge: A controlled quasi-experimental study in India and Mexico

**DOI:** 10.1371/journal.pone.0217872

**Published:** 2019-05-31

**Authors:** Yohali Burrola-Mendez, Francisco J. Bonilla-Escobar, Mary Goldberg, Jon Pearlman

**Affiliations:** 1 Department of Rehabilitation Science and Technology, University of Pittsburgh, Pittsburgh, Pennsylvania, United States of America; 2 International Society of Wheelchair Professionals (ISWP), University of Pittsburgh, Pittsburgh, Pennsylvania, United States of America; 3 Consejo Nacional de Ciencia y Tecnología (CONACYT), Ciudad de México, México; 4 SCISCO Foundation, Cali, Colombia; 5 School of Medicine, Institute for Clinical Research and Translational Science, University of Pittsburgh, Pittsburgh, Pennsylvania, United States of America; Università degli Studi di Perugia, ITALY

## Abstract

**Background:**

Evidence highlights a global shortage of wheelchair service provision education and training that results in inappropriate wheelchair provision with associated health and economic consequences. Two learning methodologies, a hybrid and an in-person course, based on the World Health Organization Wheelchair Service Training Package Basic Level, currently are available to train wheelchair service providers worldwide. The effectiveness of the in-person methodology, used as the standard of practice, has never been tested. Meanwhile, the Hybrid Course, which combines online and in-person training, was developed to reduce training costs and to scale training interventions and has shown potential effectiveness in increasing basic level wheelchair service provision knowledge. The objective of this study was to compare the effectiveness of both learning methodologies based on knowledge and satisfaction among a group of wheelchair service providers in India and Mexico.

**Methods:**

We conducted a controlled quasi-experimental study to evaluate changes in basic wheelchair knowledge and levels of satisfaction between Hybrid and In-person course learners in India and Mexico. A convenience sampling method guided by local stakeholders’ input was used to recruit participants. Outcomes were assessed using self-administered online surveys, the International Society of Wheelchair Professionals Wheelchair Service Provision Basic Test (primary outcome) completed pre- and post- the learning intervention and an anonymous Satisfaction Survey (secondary outcome) completed post- intervention. Baseline characteristics were compared among groups using hypothesis tests based on their assumptions. The primary analysis was intention-to-treat. To address missing values and lost to follow-up, multiple chained imputations were conducted. The primary outcome was analyzed using linear mixed models. The secondary outcome was analyzed using a two-tailed two independent samples t-test.

**Results:**

A total of 81 participants, 43 (53.1%) in the In-person group and 38 (46.9%) in the Hybrid group, participated in the study. Mean baseline knowledge scores were below the passing cutoff of the test (53 points) in both groups. Both study groups experienced statistically significant improvements in the primary outcome when comparing pre- and post-test scores (p<0.0001) with total mean scores **above** the passing cutoff of the test. The in-person group experienced, on average, larger effects on the primary outcome. The difference in mean change from post-test to pre-tests between In-person groups and Hybrid was 3.6 (95% Confidence Interval: 1.7;5.4), Cohen’s d = 0.36, with a small effect size favoring the In-person training. With regards to satisfaction, the difference between the two interventions was 0.23±0.07 in favor of the In-person group (p = 0.0021).

**Conclusions:**

Both learning methodologies had a statistically significant effect in increasing wheelchair service knowledge with overall high levels of satisfaction. However, the In-person group reported overall larger effects when compared with the Hybrid methodology. This study provided recommendations on how organizations can improve blended learning interventions to enhance participants’ learning experiences and reduce potential barriers and limitations.

## Introduction

The World Health Organization (WHO) estimates that only 5–15% of the 100 million people in the world who need a wheelchair for mobility and function have an appropriate wheelchair that meets their needs [1–3]. Inappropriate wheelchair provision impacts the life, safety, health, and other basic human rights of people with disabilities [2, 4–8]. In addition, when a wheelchair does not meet the wheelchair user’s needs, it may result in underutilization or abandonment [9, 10]. This situation may be more problematic in low- and middle-income countries (LMICs) where disability and poverty are interconnected, the incidence of disability is higher, people with disabilities often are marginalized, there is less availability of skilled health personnel, and there is a limited range of quality, affordable wheelchairs [1, 11–15].

Evidence highlights that a major factor associated with inappropriate wheelchair distribution is the global shortage of wheelchair service provision education and training [1, 13, 16]. The World Health Organization Guidelines on the provision of manual wheelchairs in less-resourced settings (WHO Guidelines) recommend integrating wheelchair service provision content into existing rehabilitation programs at academic institutions [2]. However, a 2017 study reported limited training time allocated to wheelchair service provision in some professional rehabilitation programs in low-middle- and high-income countries [16]. To help assess the global training need, the International Society of Wheelchair Professionals (ISWP) developed and validated a Wheelchair Service Provision Basic Test (Basic Test) which aligns with the WHO Guidelines’ eight (8) wheelchair service provision steps [17]. Currently, in the majority of regions where the test has been applied, less than half of test takers pass the test with 41% passing in Africa, 44% in Asia, 46% in Latin America, 47% in Europe, 48% in Australia and Oceania, and 55% in North America, which confirms the overwhelming need to promote training of wheelchair service providers worldwide [18].

In 2012, the WHO published the Wheelchair Service Training Package-Basic Level (WHO WSTP-B) as the first of a series of training packages, free of charge, with supporting materials and availability in different languages to promote training worldwide. The WHO WSTP-B proposes a learning methodology of 5 consecutive days of in-person training that provides the skills and knowledge for basic level wheelchair provision [19]. Traditional in-person training places a significant emphasis on human and financial resources that are often not available in resource-constrained settings [20, 21]. In addition, this learning format is difficult to scale across multiple settings, to reach underserved areas [20] and to attend for busy providers who need to leave work during the training days [22]. While this training approach has been widely used in the sector as the standard of facilitating trainings, no evidence of the effectiveness of this learning methodology has been published.

Blended learning is a cost-effective [23] and student-accepted [24–26] educational format that combines online and in-person training [27]. This type of learning has proved to be as effective as in-person learning in medical education [24, 27–29] and a feasible solution to overcome knowledge dissemination barriers in less-resourced areas [30]. In 2016, ISWP developed a Hybrid Course based on the WHO WSTP-B in English and Spanish with the scope to support efficient content delivery, decrease cost associated with leading the training, and increase access [22, 31]. The Hybrid Course uses a blended learning methodology that combines 9 online modules designed for low-bandwidth internet access which reduces the in-person training exposure to 3 days, making it easier to scale and more adaptable to different training environments such as conferences and continue educational programs at universities) [22]. The Hybrid Course has been tested in English [22] and Spanish [31] reporting a statistically significant increase on the Basic Test total score in both languages [22, 31]. While these results offer some evidence of the potential effectiveness of the Hybrid Course to train wheelchair service providers, the course has not been compared with the standard methodology of in-person training recommended by the WHO WSTP-B, nor is there evidence of the effectiveness of the in-person training approach.

The primary objective of this study was to compare the effectiveness of a Hybrid Course and In-person Course in English and Spanish in increasing knowledge in basic level wheelchair service provision. The secondary objective was to evaluate and compare levels of satisfaction with the interaction, instructors, instruction methodology, content, and technology (exclusively with the Hybrid) after the learning interventions. We hypothesized that the Hybrid Course will produce similar improvements in outcomes as the In-person Course.

## Methods

This study was a quasi-experimental design with nonequivalent control groups conducted to evaluate changes in basic level wheelchair service knowledge with a group of wheelchair service providers in Bengaluru, India and Puebla, Mexico. In each setting, one of the groups was trained using the Hybrid Course (blended methodology), and the other followed the In-person training methodology. A post-assessment was used to evaluate levels of satisfaction after the educational interventions. The study was approved by the University of Pittsburgh Institutional Review Board.

### Participants

The research team selected the countries of India and Mexico due to the presence of local facilitators, local partnerships, income classification (lower-middle- and upper-middle- income economies classified by the World Bank) [32], and the possibility to test both languages of the Hybrid and In-person courses (Table 1). Lead organizations and stakeholders used a convenience sampling method to recruit participants interested in receiving basic level wheelchair service provision training. Flyers describing the course, inclusion criteria, location, schedule, and contact information were distributed. Each organization (Table 1) led the recruitment, enrollment, and delivery of the interventions. The inclusion criteria included: 1) rehabilitation sciences students or professionals who worked locally in wheelchair service delivery, and 2) who had not taken the Basic Test. Participants who were participating in another wheelchair-related study simultaneously were excluded. The interventions occurred in different timepoints between February 2016 to February 2017 (Table 1).

**Table 1:** List of settings and lead organizations.

Country	City	Lead Organization/Partners	Intervention	Training	Language
India	Bengaluru	Mobility India	In-person course	February 2016	English
		Specialized Mobility Operations and Innovation	Hybrid course	May 2016	
Mexico	Puebla	Centro de Rehabilitación Infantil Teletón	Hybrid course	January 2017	Spanish
			In-person course	February 2017	

### Interventions

To provide a detailed description of the interventions, improve the reporting of these interventions and, ultimately their replicability, we used the Template for Intervention Description and Replication (TIDieR) checklist [33] (Table 2) and report some training costs in Table 3.

**Table 2:** TIDieR for describing the four interventions.

Intervention		ISWP Hybrid Course on Wheelchair Service Training Package—Basic Level (Hybrid)		WHO Wheelchair Service Training Package—Basic Level (In-person)	
Country		India	Mexico	India	Mexico
**Why**		Flexible and scalable learning methodologies to train wheelchair service providers need to be tested.		The in-person learning methodology to train wheelchair service providers needs to be tested.	
**What**	*Materials*	Adobe Connect, internet access, one large accessible classroom, assessment beds/mats, demo wheelchairs, donated wheelchairs, foam, participants' handbooks, participants' workbooks, trainers' manuals, training program evaluation forms, posters, chairs, wheelchairs forms, one whiteboard, computer, projector, cushion fabrication toolkits, home maintenance toolkit. The list of training resources, materials, and tools are included in the WHO Wheelchair Service Training Package Trainer's Manual Basic Level [41].		One large accessible classroom, assessment beds/mats, demo wheelchairs, donated wheelchairs, foam, participants' handbooks, participants' workbooks, trainers' manuals, training program evaluation forms, posters, chairs, wheelchairs forms, one whiteboard, computer, projector, cushion fabrication toolkits, home maintenance toolkit. The list of training resources, materials, and tools is included in the WHO Wheelchair Service Training Package Trainer's Manual Basic Level [41].	
	*Procedures*	Participants had one week prior to the beginning of the course to complete pre-assessments. The intervention consisted of two weeks of asynchronous online training (12 hours) with two or three* synchronous online meetings (recitations) (4 hours). The online training followed 3 consecutive days (24 hours) of in-person training. After this, participants had one-week to complete the post-assessments.		Participants had one week prior to the beginning of the course to complete pre-assessments. The intervention consisted of 5 consecutive days of in-person training (40 hours). After this, participants had one week to complete the post-assessments.	
**Who provided**	*Online section*	ISWP Hybrid Course developer and staff (course developer with rehabilitation research experience and background in Physical Therapy). In addition, staff available for technical problems or questions.		Not applicable	
	*In-person section*	Four trained instructors (2 Biomedical Engineers and 2 Physical Therapists)	Three trained instructors (Occupational Therapist, Medical Doctor, Biomedical Engineer)	ϕ	Three trained instructors (Occupational Therapist, Medical Doctor, Biomedical Engineer)
**Models of delivery**		One group of 19 participants was trained. During the recitations, participants interacted with each other and with instructors. In the in-person sessions, participants practiced with wheelchair users in groups of 4. (5 wheelchair users in total)	One group of 16 participants was trained. During the recitations, participants interacted with each other and with instructors. In the in-person sessions, participants practiced with wheelchair users in groups of 3. (5 wheelchair users in total)	One group of 24 participants was trained. During the in-person sessions, participants practiced with wheelchair users.^ϕ^	One group of 19 participants was trained. During the in-person sessions, participants practiced with wheelchair users in groups of 2. (10 wheelchair users in total)
**Where**		The in-person sessions were facilitated at the Association for People with Disabilities (APD) in Bangalore, India. Facilities used were two training rooms of 90 and 60 square meters respectively, and a machine shop for wheelchair adjustments and cushion fabrication.	The in-person sessions were facilitated at Centro de Rehabilitación e Inclusión Infantil Teletón in Puebla, Mexico. Facilities used were one room of 60 square meters and one machine shop for wheelchair adjustments and cushion fabrication.	The course was facilitated at Mobility India in Bangalore, India. Facilities used were one room and one machine shop for wheelchair adjustments and cushion fabrication.^ϕ^	The course was facilitated at Centro de Rehabilitación e Inclusión Infantil Teletón in Puebla, Mexico. Facilities used were one room of 60 square meters and one machine shop for wheelchair adjustments and cushion fabrication.
**When/ how much**	*Pre-assessment*	ISWP Wheelchair Service Provision–Basic Test, online test, approximately one hour to complete			
	*Online training section*	Two weeks, 9 online modules, 2–3 synchronous recitations of 60 minutes each.		Not applicable	
	*In-person training section*	Three consecutive days, 8 hours per day.		Five consecutive days, 8 hours per day.	
	*Post—assessments*	ISWP Wheelchair Service Provision–Basic Test, online test, approximately one hour to complete.			
		ISWP Hybrid Satisfaction Survey, online, approximately 30 minutes to complete.		ISWP In-person Satisfaction Survey, online, approximately 30 minutes to complete.	
**Tailoring**		The demo wheelchairs used were from the local context.			
**Modifications**		None			
**How well**	*Fidelity*	Not tested			
	*Adherence*	Overall, the intervention was delivered as planned. One participant who completed the online portion did not attend the in-person training.	Overall, the intervention was delivered as planned. One participant who completed the online portion did not attend the in-person training.	ϕ	Overall, the intervention was delivered as planned. Some wheelchair supplies we did not plan for were needed and taken from the machine shop.

* In India two recitations were facilitated; in Mexico, three.

ϕ Some details about the In-person training in India are not available due to staff turnover.

**Table 3:** Training interventions’ costs.

Country	Categories	In-person			Hybrid			Diff In-person and Hybrid^€^	% Savings^μ^
		Unit	Cost per unit*	Cost^¥^	Unit	Cost per unit*	Cost^¥^		
India	Trainers' stipend per day	5 days	600	$3,000	3 days	600	$1,800	$1,200	40%
	Staff support, hours	6 hours	30	$180	9 hours	30	$270	$-90	-50%
	Food/beverage during training, per person	24 people, 5 days	20	$2,400	21 people, 3 days	20	$1,260	$1,140	48%
	***Total***^***ϕ***^			***$5*,*580***			***$3*,*330***	***$2*,*250***	***40%***
Mexico	Trainers' stipend per day	5 days	600	$3,000	3 days	600	$1,800	$1,200	40%
	Staff support, hours	6 hours	30	$180	9 hours	30	$270	$-90	-50%
	Food/beverage during training, per person	19 people, 5 days	20	$1,900	17 people, 3 days	20	$1,020	$880	46%
	***Total***^***ϕ***^			***$5*,*080***			***$3*,*090***	***$1*,*990***	***39%***

* All costs are in the United States dollars (USD).

¥ To calculate each cost, cost per unit was multiplied by the number of units.

ϕ The *Total* is the sum of all the costs in the category.

€ This column was calculated by subtracting the categories between the In-person course and the Hybrid.

μ Represents the percentage of savings by conducting a Hybrid Course instead of an In-person course. The percentage was calculated by dividing the *Diff In-person and Hybrid* by the In-person *Cost* of the same category and then multiplying the result by 100 to obtain the percentage.

#### Hybrid course

This group followed the methodology implemented in previous studies [22, 31] that consisted of the completion of baseline assessments, two consecutive weeks of online training followed by three days of in-person training and the collection of follow-up assessments (Table 2). During the online training, participants reviewed the content and completed all required activities asynchronously that have been described elsewhere (e.g., discussion boards, case studies, short quizzes, videos, interactive activities) [22]. The online content strictly followed the WHO WSTP-B’s content and incorporated its materials (e.g., videos, Power Points) [19] whenever possible. The Hybrid Course mirrors the WHO WSTP-B training with the necessary adaptations for online learning [22]. To promote the gradual revision of online content, not all modules were accessible from the beginning of the course; instead, they were divided and made available sequentially every week. Two online synchronous meetings with the Indian groups and 3 with the Mexican groups were conducted between trainers and trainees to reinforce learning outcomes, discuss topics, answer questions, and promote interaction between participants. The Mexican groups had an additional meeting due to trainers’ availability. The recitations were mandatory, lasted 60 minutes, and were recorded and made available to all participants and trainers. In the last recitation, trainers provided detailed information about the consecutive 3-days of in-person training led by their facilitating organization.

#### In-person course

This group followed the learning methodology of 5 consecutive days of in-person training, 8 hours per day, described in the WHO WSTP-B Trainers Manual [34]. Theoretical and practical sessions occurred simultaneously. Trainers used the WHO WSTP-B materials (e.g., videos, Power Points, assessment forms) to facilitate the training.

In both groups, a local ‘master trainer’ coordinated the trainings. A ‘master trainer’ was considered someone who had been trained with the WHO WST-B previously, who had passed the ISWP Basic Test, and had experience facilitating WHO WSTP-B courses. Also, in both groups, wheelchair users volunteered in the training as role models which allowed participants to directly interact with them (Table 2). In addition, all groups delivered basic level wheelchairs to wheelchair users at the end of the training following the WHO 8-steps learned in the education interventions.

### Outcomes

To be consistent with the previous studies that evaluated the effectiveness of the Hybrid Course [22, 31], we established knowledge change as our primary outcome measure and levels of satisfaction as our secondary measure. These two outcome measures are relevant to evaluate the influence of both learning methodologies in knowledge change and the courses' acceptance among trainees.

The Basic Test is an online test available in English and Spanish, that has shown validity evidence for measuring basic level wheelchair service provision knowledge independent of geographic location [17]. The test consists of two sections: A demographic questionnaire and a multiple-choice test. The demographic questionnaire includes 19 questions regarding sociodemographic characteristics of participants such as age, gender, education level, profession, employment status, years of experience in wheelchair service provision, work setting, age group served, and motivation to take the training. In the questions related to the work setting, age group served, and motivation to take the training, participants can select all applicable options. The multiple-choice test includes 75 scored questions from 7 domains of wheelchair service delivery knowledge: 1) assessment, 2) prescription, 3) fitting, 4) production, 5) user training, 6) process and 7) follow-up and maintenance as described in the WHO WSTP-B [17]. The test settings include: 1) a pre-set number of questions based on the weight of each domain that is obtained from a pool of questions to reduce the likelihood of receiving the same questions in multiple attempts; 2) a forced completion in one-time entry; and 3) immediate test score reporting with the opportunity to review correct and incorrect answered questions [17, 22, 31]. Test scores greater than or equal to 53 points, or 70% of correctly answered questions, are considered passing scores.

The ISWP Hybrid Satisfaction Survey (Hybrid Survey) is an online questionnaire available in English and Spanish that evaluates levels of satisfaction among participants after the learning intervention [31]. The Hybrid Survey is integrated by 5 sub-domains: Interaction, instructor, instruction methodology, content and technology and uses a five-point Likert scale (4 = strongly agree, 3 = agree, 2 = neither agree nor disagree, 1 = disagree, 0 = strongly disagree) for participants to indicate the degree to which they agree with each statement. Open-ended questions at the end of each sub-domain asked participants to provide suggestions and feedback [31]. We created the ISWP In-person Satisfaction Survey (In-person Survey) from the existing Hybrid Survey by (1) removing the questions related the online component of the course through the sub-domains, and (2) eliminating the technology sub-domain (S1 File).

In both groups, participants were instructed to complete the Basic Test without accessing course materials one-week pre-post the learning intervention. The test was hosted in a testing platform, Test.com, and completed online. Participants received an email with instructions on how to log into Test.com and the contact information for ISWP’s staff in case of technical problems or questions. In addition, participants were encouraged to complete the ISWP Hybrid or In-person Satisfaction Survey anonymously one week post the learning intervention. The Surveys were hosted online in Qualtrics and distributed via an external link to all participants. The Indian groups completed all outcome measures in English, while the Mexican groups did so in Spanish.

### Sample size

The intended group size for each intervention was determined to be between 15–20 participants based on the trainer-trainee ratio suggested by WHO WSTP-B to promote an appropriate learning environment since the program had a significant amount of hands-on practical sessions [34]. We estimated the power of the study using information from the analyzed data with the command to estimate power for a two-sample means test clustered design.

### Assignment method

Interventions were facilitated at different timepoints, and each training followed its own convenience sampling method. The training interventions were facilitated at no cost to participants; hence, to reduce attrition, the participant’s supervisor’s approval was necessary to enroll in the study. During the interventions, the study outcome measures were self-administered online at specific timepoints (Table 2). Participants’ completion of the Basic Test was continuously monitored by the research team and reported to facilitating organizations. Trainers sent reminders via email and followed up with participants when a trainee did not complete the test within the given timeframe. For the secondary outcome measure, due to the anonymity of the survey, individual follow-up was not feasible. Nevertheless, trainers encouraged participants in the last day of the training to complete the surveys and provide feedback.

### Masking

Participants, trainers, staff, and the research team were not masked to the study learning intervention assignment.

### Unit of analysis and statistical analysis

Treatment impact was derived using longitudinal modeling of within-person change in mean scores of the basic test knowledge from baseline (pre-test) to follow-up (post-test). Analysis of satisfaction was limited to trainees’ follow-up responses.

Baseline characteristics of participants from intervention and control groups were compared using Chi-square or two-tailed Fisher’s exact test for categorical variables and t-student or Wilcoxon test for continuous variables after assessing the test assumptions. Outliers were considered extreme values equal to or greater than 4 standard deviations. The indicator of effectiveness in increasing basic wheelchair service provision knowledge was derived from comparing mean changes in the total test scores from baseline to follow-up assessment. These differences were compared again between each other to assess the differences in the effectiveness of the Hybrid and In-person groups.

The design of the study was intended to analyze the effects of the interventions in both countries simultaneously. The trainee was the unit of analysis. The primary outcome was the change in the basic knowledge test score measured by the Basic Test; the secondary outcome was the satisfaction level measured by the Hybrid/In-person Satisfaction Survey. All outcomes were treated as continuous variables.

#### Primary outcome analysis: Knowledge test scores

A mixed effects model, with a robust estimate of variance, was used to estimate the effect of training strategies (Hybrid or In-person) including time point (0 = pre-test; 1 = post-test) and participant ID as random effects to account for within-person correlation across time and between-person correlation. This assessed the mean difference between intervention conditions (Hybrid vs. In-person training) in changes in knowledge score over time within both Hybrid and In-person training.

Lost to follow up was handled using two methods: 1) multiple chained-imputations of covariates and scores for those lost to follow up and 2) a sensitivity analysis creating an inverse probability weight of follow up and including this weight as a covariate in the sensitivity models. Missing values including follow-up scores for those lost to follow-up in the knowledge test were handled using chained equations command for multiple imputations in Stata, which pools data according to Rubin's rules [35, 36]. We assumed missing at random (MAR) for the imputation model and following a methodology described by Bolton et al [37, 38], we first imputed any missing data on demographic variables based on all other demographic variables and educational strategy. A total of 11 imputations were used. Baseline and follow-up knowledge scores on all items missing data were then imputed using all variables in the dataset. Educational strategies were imputed separately. Sum scores based on the seven domains of the Basic Test were then calculated in the multiple imputation framework using all imputed datasets to acquire the final test score. We did not perform any data transformation. All final outcome models were run across the 11 imputed datasets.

Statistical significance was set at a 0.05 alpha level, two-tailed, and expressed as a 95% confidence interval. Cohen's d effect sizes were calculated by dividing the difference in average change from baseline to follow-up between the Hybrid and In-person groups by the outcome's pooled standard deviation at baseline. Between group effect sizes were calculated using Cohen’s d statistic. Effect sizes of 0.2 were considered small, 0.5 medium, and 0.8 or above large.[39] All analyses used the full intent-to-treat (ITT) sample.

#### Adjusted models

An ITT analysis, which included all study participants based on participants’ group allocation with the multiple imputations database, was used to mitigate the effects of loss of follow-up. Outcomes were adjusted to account for possible residual confounding. Co-variables included in final models were those that were significant at the p<0.10 level identified using: 1) simple logistic regression clustering by country and participant to identify baseline differences between interventions; and 2) mixed models to determine interactions between potential co-variables and time on knowledge test scores. Furthermore, models were adjusted for age, gender, and education, which are well-known confounders of the relationship between the intervention and outcome in educational research. All possible confounding variables (both dichotomous and continuous) were centered in order to report the averaged sample effect of the interventions. Multicollinearity was explored using the variance inflation factor (VIF) considering values of VIF>5 indications of collinearity between variables. These variables were removed from the models. To explore whether the country should be treated as a random or fixed effect, a Hausman test was utilized [40].

The model for total test scores was adjusted by age, gender, educational level, work setting, student or professional status, and baseline domain test scores. The models for gender categories were adjusted by age, educational level, work setting, student or professional status, and baseline domain test scores. The models for age categories were adjusted by gender, educational level, work setting, student status, and baseline domain test scores. The models for education level categories were adjusted by age, sex, work setting, and baseline domain test scores. The models for wheelchair service provision experience categories were adjusted by age, gender, educational level, work setting, student or professional status, and baseline domain test scores. The country also was included in all models as a fixed effect, as the Hausman test was significant (p<0.0001) [40]. To test for the effect of outliers, we were planning to exclude them from the analyses and run new models without outliers, but there were no outliers in the test scores.

#### Secondary outcome analysis: Levels of satisfaction

Q-Q plots were used to assess the normal distribution of the data. The Variance Ratio Test (sdtest in Stata) was used to assess homoscedasticity. Survey responses were analyzed using means and standard deviations. Survey domains scores were obtained by summing the type of response selected (4 = strongly agree, 3 = agree, 2 = neither agree nor disagree, 1 = disagree, 0 = strongly disagree) dividing them by the total number of respondents and then obtaining the standard deviation. A total satisfaction mean was obtained by calculating the mean per subject using a pre-selected set of 15 questions that did not vary across the Hybrid/In-person Satisfaction surveys.

To assess differences in satisfaction between the groups, we tested if the individuals and country have an additional effect on the outcome. Using the generalization of the Hausman test in Stata, we found that the effect of the individuals and country was not significant. Additionally, we tested if the country as a cluster would have an impact on the analysis using the intraclass correlation coefficient (ICC) for the country. To obtain the ICC, we developed a mixed regression model using the following variables: Total satisfaction means as the dependent variable, Hybrid or In-person group as an independent variable, and the country as a cluster. The calculated ICC was close to zero (3.541^−48^); therefore, clustering by country was disregarded. A two-sample t-test with equal variances was used to describe the comparison on satisfaction between the two groups (Hybrid and In-person) considering a significant difference a p-value <0.05.

## Results

### Sample analyzed

**Fig 1:**
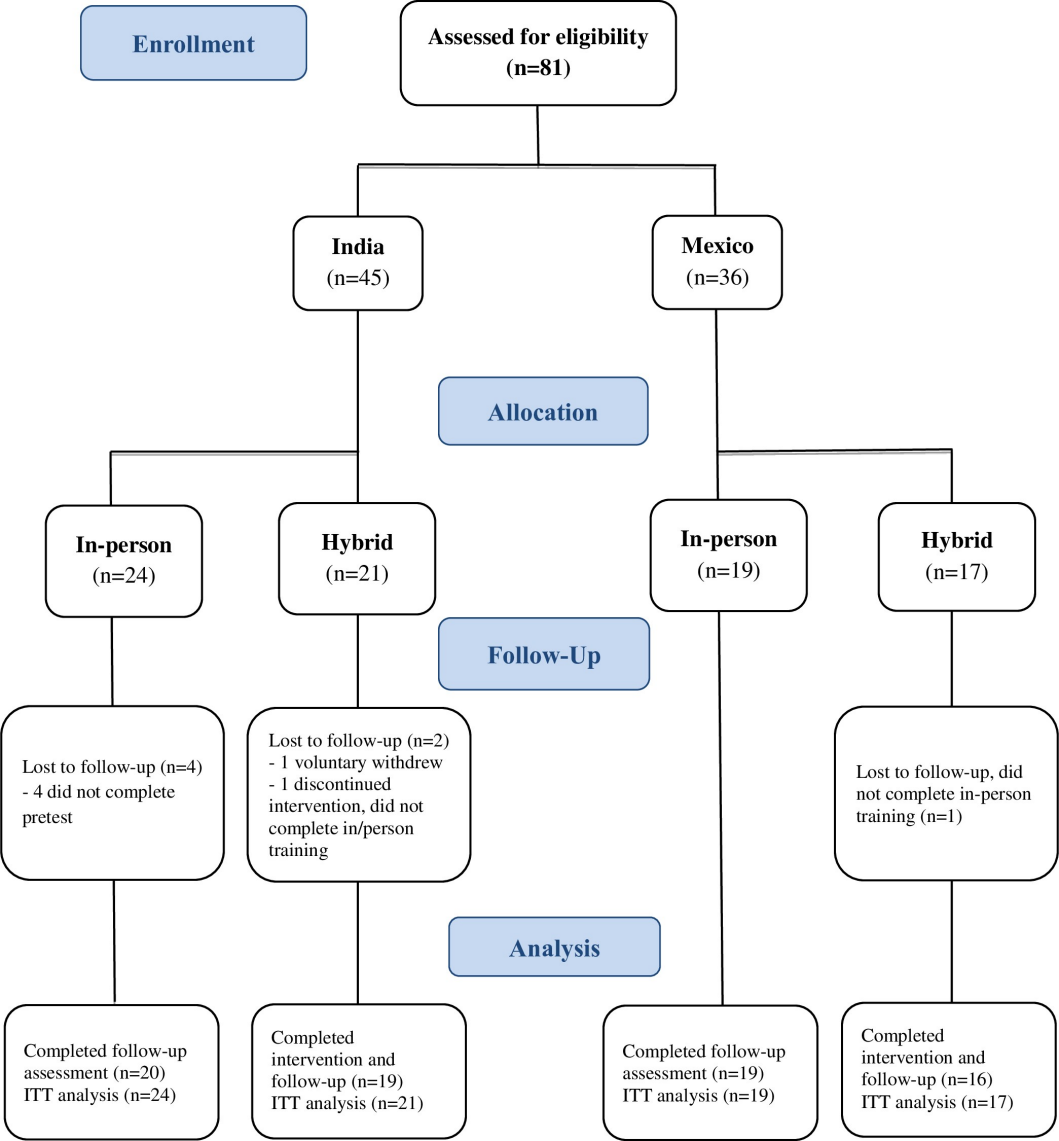
Flow chart of study participants.

A total of 81 eligible participants were recruited across countries to participate in the study (n = 45 in India and n = 36 in Mexico) from February 2016 to February 2017. In India, 24 (53.3%) participants formed the In-person group and 21 (46.6%) the Hybrid group. In Mexico, 19 (52.8%) participants formed the In-person group and 17 (47.2%) the Hybrid group (Table 4). A total of 38 (46.9%) participants were enrolled in the Hybrid course while 43 (53.1%) were enrolled in the In-person course. In the In-person group, 4 participants were lost to follow-up (India), while in the Hybrid groups, 1 participant voluntarily withdrew from the intervention (India), and 2 were lost to follow-up (India and Mexico) (Fig 1).

**Table 4:** Characteristics of participants and baseline scores.

Characteristics	India								Mexico		
	In-person (n = 24)				Hybrid (n = 21)			p-value	In-person (n = 19)	Hybrid (n = 17)	p-value
**Gender, n (%)**								0.69^a^			1.00^c^
Men	14 (56)				11 (44)				3 (60)	2 (40)	
Women	10 (50)				10 (50)				16 (51.6)	15 (48.4)	
**Age, mean (SD)**	36 (1.6)				30 (1.3)			0.007 ^b^	35.8 (18.9)	23.5 (1.2)	<0.0001^b^
**Educational level, n (%)**								0.76^c^			<0.0001^c^
<Bachelor	4 (66.7)				2 (33.3)				2 (13.3)	13 (86.7)	
Bachelor	8 (57.1)				6 (42.9)				12 (80)	3 (20)	
Graduate degree or more	12 (48)				13 (52)				5 (83.3)	1 (16.7)	
**Last year of formal training, n (%)**								0.01^c^			0.003^a^
Still attending	1 (12.5)				7 (87.5)				2 (15.4)	11 (84.6)	
<4 years	8 (47.1)				9 (52.9)				8 (72.7)	3 (27.3)	
4 years or more	15 (75)				5 (25)				9 (75)	3 (25)	
**Previous wheelchair training, n (%)**								0.14^a^			0.33^a^
No	16 (47.1)				18 (52.9)				12 (60)	8 (40)	
Yes	8 (72.7)				3 (27.3)				7 (43.8)	9 (56.2)	
**Student, n (%)**								0.02^c^			<0.0001^c^
No student	24 (60)				16 (40)				17 (77.3)	5 (22.7)	
Yes student	0 (0)				5 (100)				2 (14.3)	12 (85.7)	
Physical therapy	0 (0)				4 (100)				0 (0)	8 (100)	
Occupational therapy	0 (0)				0 (0)				2 (33.3)	4 (66.7)	
Other	0 (0)				1 (100)				0 (0)	0 (0)	
**Professional, n (%)**								0.001^c^			<0.0001^c^
No professional	0 (0)				7 (100)				2 (14.3)	12 (85.7)	
Yes professional	24 (63.2)				14 (36.8)				17 (77.3)	5 (22.7)	
Physical therapist	8 (42.1)				11 (57.9)				6 (100)	0 (0)	
Occupational therapist	10 (90.9)				1 (9.1)				7 (70)	3 (30)	
Prosthetics and Orthotics	3 (75)				1 (25)				0 (0)	0 (0)	
Physiatry (MD)	0 (0)				0 (0)				4 (80)	1 (20)	
Other	3 (75)				1 (25)				0 (0)	1 (100)	
**Employment status, n (%)**								0.08^c^			<0.0001^c^
Unemployed	0 (0)				4 (100)				1 (10)	9 (90)	
Half time	2 (50)				2 (50)				0 (0)	2 (100)	
Full time	22 (59.5)				15 (40.5)				18 (75)	6 (25)	
**Work setting: yes, n (%)**											
Hospital	15 (65.2)				8 (34.8)			0.10^a^	9 (90)	1 (10)	0.008^c^
Academic	11 (68.8)				5 (31.2)			0.12^a^	1 (8.3)	11 (91.7)	<0.0001^a^
Outpatient	7 (41.2)				10 (58.8)			0.20^a^	6 (42.9)	8 (57.1)	0.34^a^
In-patient	4 (44.4)				5 (55.6)			0.71^c^	4 (50)	4 (50)	1.00^c^
**Age group served: yes, n (%)**											
Children	7 (58.3)				5 (41.7)			0.69^a^	12 (70.6)	5 (29.4)	0.04^a^
Adolescent	8 (57.1)				6 (42.9)			0.73^a^	4 (40)	6 (60)	0.46^c^
Adults	18 (52.9)				16 (47.1)			0.93^a^	8 (42.1)	11 (57.9)	0.18^a^
Older adults	7 (43.8)				9 (56.2)			0.34^a^	2 (50)	2 (50)	1.00^c^
**Motivation training: yes, n (%)**											
Professional growth	19 (47.5)				21 (52.5)			0.05^c^	19 (57.6)	14 (42.4)	0.09^c^
Personal growth	6 (75)				2 (25)			0.25^c^	2 (33.3)	4 (66.7)	0.39^c^
Required by academic program	5 (100)				0 (0)			0.05^c^	1 (12.5)	7 (87.5)	0.02^c^
**Wheelchair service provision experience, years, n (%)**								0.54^c^			0.45^c^
<3 years	14 (46.7)				16 (53.3)				13 (46.4)	15 (53.6)	
4–7 years	6 (66.7)				3 (33.3)				3 (75)	1 (25)	
8 years or more	4 (66.7)				2 (33.3)				3 (75)	1 (25)	
**Wheelchair service provision, hours, n (%)**								0.91^c^			0.35^c^
<3 hours/week	10 (58.8)				7 (41.2)				10 (47.6)	11 (52.4)	
3–19 hours/week	11 (50)				11 (50)				6 (50)	6 (50)	
20 hours/week or more	3 (50)				3 (50)				3 (100)	0 (0)	
**Member of an organization: yes, n (%)**	19 (61.3)				12 (38.7)			0.11 ^a^	14 (70)	6 (30)	0.02^a^
**Mean Scale scores, mean (SD)**											
Total Wheelchair Service Basic Test *	44.8 (8.6)				44.6 (6.3)			0.92^b^	42.9 (9.7)	40.8 (8.6)	0.50^b^
Assessment	13.5 (2.4)				12.5 (2.3)			0.15^b^	13.2 (3.5)	12.7 (3.1)	0.65^b^
Prescription	6.6 (2)				6.5 (2)			0.90^b^	7.1 (1.9)	7.1 (1.5)	0.98^b^
Fitting	3.9 (1.8)				4.2 (1.9)			0.61^b^	3.8 (1.7)	3.6 (1.2)	0.69^b^
Production	3 (1.5)				3 (1.3)			0.91^b^	2.4 (1.3)	2 (1.4)	0.43^b^
User’s training	8.5 (2.3)				8.8 (1.8)			0.63^b^	7.9 (2.5)	7 (2)	0.25^b^
Process	6.8 (2.1)				6.9 (1.5)			0.92^b^	6.2 (3.4)	5.9 (2.5)	0.75^b^
Follow up and maintenance	2.4 (1)				2.7 (1)			0.51 ^b^	2.3 (1.2)	2.5 (1.3)	0.61^b^

SD: Standard deviation

^a^ Chi-square test

^**b**^ t test

^**c**^ Fisher's exact test

^**d**^ Wilcoxon rank-sum

* All total Wheelchair Service Basic Test scores, not paired.

### Sociodemographic and baseline characteristics

In India, sociodemographic characteristics between In-person and Hybrid groups were similar except for age, last year of formal training, and student status. In the Hybrid group, the group was significantly younger (Mean (M) = 30, Standard Deviation (SD) = 1.3), had more students (5/21, 23.8%) and more participants with less than 4 years of experience (n = 16, 76.2%) than in the In-person group. In Mexico, fewer sociodemographic characteristics were similar between In-person and Hybrid groups. Overall, the Hybrid group was significantly younger (M = 23.5, SD = 1.2) and comprised of mostly students (12/17, 70.6%). This situation translated into statistically significant baseline differences in other variables such as educational level, last year of formal training, employment status, work settings, and motivation to take the training. Despite the baseline difference, the Basic Test total scores and domain scores were similar between Hybrid and In-person groups in both countries (Table 4).

### Primary outcome: Basic level wheelchair knowledge

A paired sample t-test indicated that post assessment total scores were significantly higher after the training experience in the In-person and Hybrid group of both countries (Table 5). All domain scores mean values increased after the training interventions. The domains that did not report statistically significant changes in India’s In-person group were “Production” and “Follow up and maintenance”; while Mexico’s In-person was “Follow up and maintenance.” In India’s Hybrid group, “Fitting” and “Production” did not report statistically significant changes. In contrast, all domains in Mexico’s Hybrid group reported statistically significant changes (Table 5).

**Table 5:** Paired sample test scores of knowledge based on the ISWP basic test.

Mean Scale scores, mean (SD)	India						Mexico					
	In-person (n = 20)*		p-value	Hybrid (n = 19)		p-value	In-person (n = 19)		p-value	Hybrid (n = 16)		p-value
	Pre	Post		Pre	Post		Pre	Post		Pre	Post	
Total Score, mean (SD)	44.8 (8.6)	59.7 (9.4)	<0.0001	45.2 (6.3)	57.8 (7.3)	<0.0001	42.9 (9.7)	58.8 (6.5)	<0.0001	41.3 (8.7)	55 (5.6)	<0.0001
Assessment	13.5 (2.4)	16.5 (2.7)	0.0009	12.6 (2.4)	16 (0.7)	<0.0001	13.2 (3.5)	17 (1.5)	<0.0001	13.1 (2.8)	16.1 (1.9)	0.0009
Prescription	6.6 (2)	10.6 (1.3)	<0.0001	6.7 (1.9)	9.5 (1.3)	<0.0001	7.1 (1.9)	9.5 (1.4)	0.0001	7.2 (1.5)	9.9 (1.3)	<0.0001
Fitting	3.9 (1.8)	5.7 (2.1)	0.0010	4.4 (1.9)	5.5 (1.7)	0.060	3.8 (1.7)	5.3 (1.6)	0.0017	3.6 (1.2)	4.8 (1.8)	0.044
Production	3 (1.5)	3.8 (1.3)	0.066	3 (1.3)	3.6 (1.3)	0.134	2.4 (1.3)	3.9 (0.9)	0.0005	1.9 (1.4)	3.3 (1)	0.0028
User’s training	8.5 (2.3)	11.6 (2.2)	<0.0001	8.8 (1.9)	11.1 (1.8)	0.0017	7.9 (2.5)	11.7 (1.6)	<0.0001	7.1 (2.1)	9.2 (1.9)	0.024
Process	6.8 (2.1)	8.6 (1.5)	0.0035	6.9 (1.5)	8.5 (1)	0.0024	6.2 (3.4)	8.3 (1.9)	0.0231	5.9 (2.6)	8.3 (1.1)	0.0012
Follow up and maintenance	2.4 (1)	2.8 (1.1)	0.246	2.7 (1.1)	3.4 (0.8)	0.0175	2.3 (1.2)	2.9 (1.3)	0.1105	2.5 (1.3)	3.4 (0.7)	0.0267

*missing data in 4. SD = Standard deviation

#### Effectiveness of the intervention

**Fig 2:**
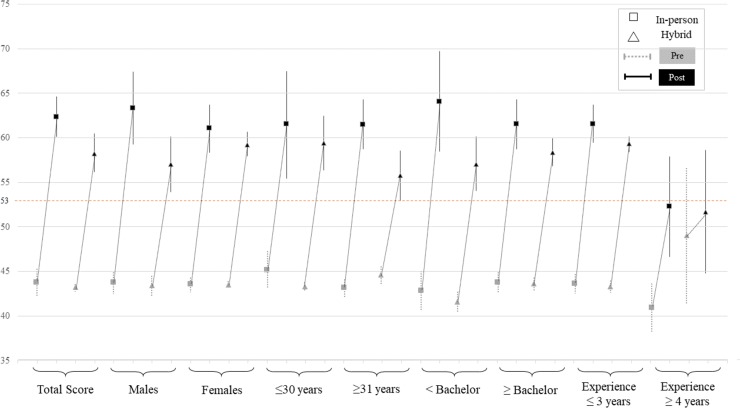
Adjusted pre- and post-test scores mean with their 95%confidence intervals by type of intervention.

Table 6 presents the adjusted intervention effects of overall Basic Test in all the participants and across sub-groups including the following scores: Pre, post, the difference between pre and post, and the interventions’ difference obtained when comparing the differences of In-person and Hybrid courses (difference of differences). Both study groups experienced statistically significant improvements in the primary outcome when comparing post- and pre-test scores (p<0.0001). When the primary outcome was analyzed by subgroups, statistically significant increases were found in all subgroups except on providers with ≥4 years of wheelchair service experience (p = 0.091) in the Hybrid group. The In-person group experienced, on average, larger effects on the primary outcome. Statistically significant differences favoring the In-person group were founded in overall total test scores (p<0.0001, d = 0.36, small effect), and total test scores sub grouped by male (p = 0.001 d = 0.48, small effect), age ≥31 years (p = <0.0001, d = 1.02, large effect), educational level ≥bachelor (p = 0.002, d = 0.50, moderate effect) and wheelchair service provision experience ≥4 years (p = 0.001, d = 0.69, large effect) (Table 6 and Fig 2).

**Table 6:** Effectiveness of In-person and hybrid interventions.

Characteristics, mean, (95% CI)	Measure	In-person	Hybrid	p-value	Effect size
**Test scores**	Pre	43.7 (42.2; 45.3)	43.2 (42.7;43.6)		
	Post	62.4 (60.2;64.7)	58.2 (56.1;60.4)		
	Post-Pre [p]	18.7 (15.1;22.2) [<0.0001]	15.1 (12.7;17.4) [<0.0001]		
	Diff	-3.6 (-5.4; -1.7)		<0.0001	0.36
**Gender**					
Male	Pre	43.7 (42.4; 44.9)	43.4 (42.2; 44.5)		
	Post	63.4 (59.3; 67.5)	57 (53.9; 60.1)		
	Post-Pre [p]	19.7 (14.9; 24.6) [<0.0001]	13.7 (11.4; 15.9) [<0.0001]		
	Diff	-6.1 (-9.5; -2.6)		0.001	0.48
Female	Pre	43.5 (42.6; 44.3)	43.5 (43.2; 43.9)		
	Post	61.1 (58.4; 63.8)	59.2 (57.9; 60.6)		
	Post-Pre [p]	17.6 (14.3; 21) [<0.0001]	15.7 (14.4; 17) [<0.0001]		
	Diff	-1.9 (-4.2;0.4)		0.10	0.23
**Age**					
≤30 years	Pre	45.1 (43; 47.3)	43.3 (42.8; 43.8)		
	Post	61.6 (55.5; 67.6)	59.4 (56.3; 62.4)		
	Post-Pre [p]	16.4 (10.4; 22.5) [<0.0001]	16.1 (13.1;19) [<0.0001]		
	Diff	-0.3 (-4.3;3.6)		0.86	0.02
≥31 years	Pre	43.1 (42.1; 44.1)	44.6 (43.6; 45.6)		
	Post	61.5 (58.5; 64.4)	55.7 (52.9; 58.5)		
	Post-Pre [p]	18.4 (16;20.8) [<0.0001]	11.1 (8.9;13.3) [<0.0001]		
	Diff	-7.2 (-11.2; -3.3)		<0.0001	1.02
**Education level**					
< bachelor	Pre	42.8 (40.6; 45)	41.6 (40.5; 42.7)		
	Post	64.1 (58.5; 69.8)	57 (54; 60.1)		
	Post-Pre [p]	21.3 (13.7;28.9) [<0.0001]	15.4 (12.7;18.2) [<0.0001]		
	Diff	-5.9 (-10.9; -0.9)		0.22	0.31
≥bachelor	Pre	43.7 (42.6; 44.9)	43.6 (42.8; 44.3)		
	Post	61.6 (58.8; 64.4)	58.3 (56.8; 59.9)		
	Post-Pre [p]	17.8 (15.4;20.3) [<0.0001]	14.8 (13.5;16) [<0.0001]		
	Diff	-3.1 (-4.8; -1.3)		0.002	0.50
**WC provision experience**					
≤ 3 years	Pre	43.6 (42.5; 44.7)	43.3 (42.6; 44)		
	Post	61.6 (59.5; 63.8)	59.3 (58.4; 60.1)		
	Post-Pre [p]	18.1 (14.9;21.2) [<0.0001]	15.9 (14.7;17.2) [<0.0001]		
	Diff	-2.1 (-4.6;0.4)		0.1	0.28
≥4 years	Pre	40.9 (38.2; 43.6)	49.1 (41.4; 56.7)		
	Post	52.3 (46.6; 57.9)	51.6 (44.7; 58.6)		
	Post-Pre [p]	11.3 (6.6;16) [<0.0001]	2.6 (-0.4;5.6) [0.091]		
	Diff	-8.7 (-13.7; -3.8)		0.001	0.69

Mixed effect clustered models with multiple chained imputations. All models were adjusted by age, gender (except in the subgroup analysis of gender), educational level (except in the subgroup analysis of educational level), work setting, baseline domain test scores. Interactions between timepoint and professional category (except in the subgroup analysis of educational level), educational level, and member of the organization. The professional category was included in all models except in age and educational level subgroup models due to an inflated VIF (14). Student category was excluded in educational level subgroup models due to an inflated VIF (6). 95% CI: 95% Confidence Interval. WC: wheelchair. Pre: pre-test score, mean. Post: post-test score, mean. Post-Pre: Difference from post and pre-test scores. Diff: Difference in adjusted mean score change, mean. The [p] stands for the p-value of the means comparison between post and pre. The effect size between groups was measure by Cohen’s d ([mean group 1- mean of group]/pooled standard deviation of the two groups). Effect size interpretation: small effect (0.20–0.49), moderate effect (0.50–0.79), large effect (≥0.80).

#### Sensitivity analyses

The sensitivity analysis did not show changes in the significance of the differences of differences between the Hybrid and In-person groups in the total knowledge score nor in the subgroup analyses.

### Secondary outcome: Levels of satisfaction

A total of 71 Satisfaction Surveys were collected, 41 (50.6%) from the In-person group and 30 (37%) from the Hybrid group. The means and standard deviations of the In-person and Hybrid Surveys’ domains are in Table 7.

**Table 7:** In-person and Hybrid mean and standard deviation total scores.

Domains, Mean (SD)*	In-person, n = 41	Hybrid, n = 30
Interaction	3.78, (0.30)	3.48 (0.74)
Instructor	3.90 (0.23)	3.57 (0.76)
Instruction Methodology	3.80 (0.27)	3.65 (0.37)
Content	3.81 (0.30)	3.53 (0.43)
Technology	-	3.12 (0.62)

*maximum score is 4 points.

**Fig 3:**
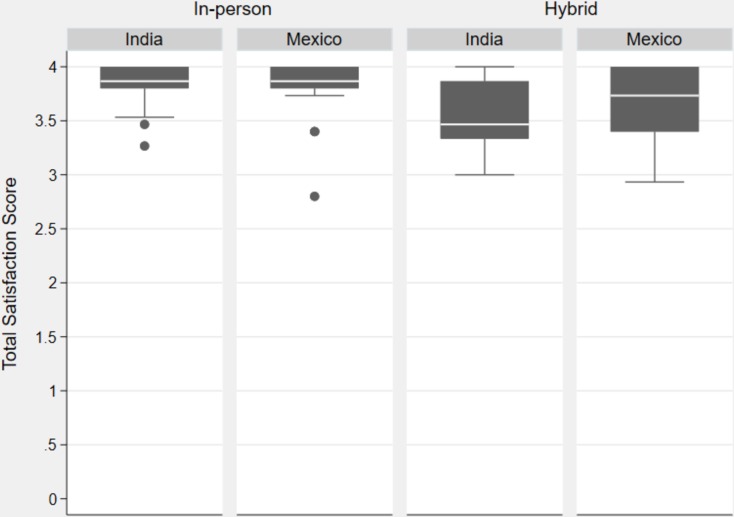
Box plots of satisfaction mean scores by type of intervention and country.

Table 8 presents the total means and standard deviations of the pre-set of questions analyzed from both surveys and Fig 3 depicts box plots of satisfaction mean scores of the same pre-set of questions by type of intervention and country.

**Table 8:** Means and standard deviations of questions analyzed.

Domain, Mean (SD)	Question	In-person (n = 40)*	Hybrid (n = 28)*, Mean, SD
Interaction	I am satisfied with the quality of interaction between all involved parties (instructor and participants).	3.78 (0.42)	3.63 (0.81)
	I am satisfied with the way I interacted with other students.	3.56 (0.71)	3.50 (0.86)
Instructor	I was satisfied with the accessibility and availability of the instructor(s).	3.95 (0.22)	3.63 (0.80)
	I continuously received feedback throughout this course.	3.85 (0.36)	3.47 (0.82)
Instruction Methodology	After this course, my understanding of wheelchair service provision has improved.	3.95 (0.22)	3.90 (0.30)
	I am satisfied with the level of effort this course required.	3.90 (0.30)	3.63 (0.49)
	I am satisfied with my performance in this course.	3.71 (0.51)	3.47 (0.57)
	I am satisfied with how I will be able to apply what I have learned in this course.	3.66 (0.58)	3.62 (0.56)
	I enjoyed this course.	3.98 (0.16)	3.77 (0.43)
Content	The goals of this course were clearly stated at the beginning of the course.	3.80 (0.46)	3.47 (0.51)
	My expectations for this course were met.	3.78 (0.42)	3.47 (0.57)
	In my opinion, the objectives of this course have been accomplished.	3.80 (0.40)	3.45 (0.51)
	Other reading materials assigned were relevant to the course objective.	3.70 (0.52)	3.63 (0.49)
	Overall, the content of the videos was relevant to the learning outcomes of the course.	3.83 (0.44)	3.38 (0.73)
	I am satisfied with this course and will recommend it to others.	3.90 (0.30)	3.75 (0.44)

*missing data in 1 (In-person) and 2 (Hybrid).

Total satisfaction in the In-person course was 3.81 (SD 0.25) while for the Hybrid course it was 3.58 (SD 0.35). The difference between the two interventions was 0.23 in favor of the In-person group (p = 0.0021).

Open-ended questions were analyzed individually and some of the comments received at the end of each sub-domain are presented in Table 9. The Hybrid groups provided more observations than the participants from the In-person groups. Overall, Hybrid participants reported technological problems when trying to watch the videos and suggested using programs that allow video streaming in places with low to medium internet speed. The alternative methods implemented by the trainers to mitigate the problems, such as pdfs and screenshots, were reported useful.

**Table 9:** Some participants’ comments from the In-person and Hybrid courses.

Domains	In-person*	Hybrid
Interaction	Excellent course we just need to practice more how to use some tools. *Participant from Mexico*	Learning the materials in advance through online modules kept us prepared for our practicals in the in-person training …At the end of session we went confidently because we gave actual wheelchair services to real patients. The instructor was very helpful at all points especially for me being in rural India the online modules wouldn't work well with the speed we have here but she sent pdfs of contents and quizzes. *Participant from India* Please reduce the video size it was difficult to play. *Participant from India*
Instructor	The instructor provided adequate guidance. *Participant from Mexico*	The instructor was always available during the training through email, phone, and in person. *Participant from India* Instructors were well prepared and provide feedback throughout the course. *Participant from Mexico*
Instruction methodology	I enjoyed the group activities and dynamics. *Participant from Mexico*	I enjoyed the Hybrid approach and looking forward to another training in the future. *Participant from Mexico* The training improved my understanding of wheelchair provision thousand times. *Participant from India*
Content		The video was buffering, and it was difficult for me to answer the video activities. *Participant from India* The videos were not loading properly even with good internet connection. *Participant from India*
Technology		Technical problems may be because of my low internet but thanks for sending screenshots of the videos… I suggest using another application that will run well with moderate speed internet. The videos were buffering a lot to play… consumes more time. *Participant from India*

*In Mexico, only the In-person group submitted comments, the Hybrid did not.

## Discussion

### Summary of results

This project demonstrated that the Hybrid Course and In-person Course were effective in increasing basic level wheelchair service knowledge with overall high levels of satisfaction among a group of rehabilitation students and professionals in India and Mexico. The results of this study help building confidence in the applicability and effectiveness of the lower-cost and more scalable Hybrid approach to training wheelchair service providers. Despite the In-person course had, on average, higher total test scores and levels of satisfaction this methodology showed only a small effect size superior to the hybrid methodology. The lessons learned from this study could help organizations to improve blended learning interventions and enhance participants’ learning experiences.

### Differences between the Hybrid and In-person groups

The Hybrid and In-person training interventions had a statistically significant influence on the total Basic Test score when comparing post- and pre-test scores (p<0.0001) which make both interventions effective in increasing basic level wheelchair service knowledge. These results are consistent with other studies conducted in LMICs in which blended learning interventions proved to be as effective as in-person interventions [20, 24, 41–43]. However, the In-person group experienced, on average, larger effects on the total test scores and on the subgroup analysis. This finding is consistent with the study results reported by Vichitvejpaisal et al. [44]. In their study, a prospective randomized design, medical students in a traditional methodology group reported better in the short-term compared to a group using computer-assisted instruction [44].

A possible explanation of our study’s In-person larger effects could be related to technological problems faced by Hybrid learners. The technology domain of the Hybrid Satisfaction Survey reported the lowest satisfaction when compared with the other domains (Table 7). Unfortunately, the survey did not identify problems encountered by participants, nor if they could or could not resolve them. Nevertheless, the comments received in the open-ended section of the survey point out that participants from the Hybrid group in India had issues accessing the modules and watching the videos due to limited internet access; some comments suggested that the actions implemented by the trainers (i.e., sending screenshots and pdfs) were effective. Previous studies have noted that effectiveness of blended education can be diluted by technological barriers such as limited access to digital technology (e.g., inadequate computer facilities, limited access to computers) [24, 30, 45], computer illiteracy [30], and limitations in bandwidth which often contributed to slow speed and low quality of videos or visual outputs [24, 30, 46–49]. Strategies that can combat these challenges include developing access hubs at strategic central locations to provide the required technology and internet access [30, 50]; developing offline content delivery platforms to overcome slow internet connectivity [30, 50], developing courses using guidelines for low bandwidth design [51] and limited technological demands in order to be more adaptable [27, 52, 53]. Another common barrier is the inadequate technological support of blended programs [45].

To some extent, our program implemented strategies to reduce barriers such as: 1) a repository of offline videos and pdfs distributed to trainers; 2) the use of a Hybrid Course developed considering low bandwidth internet access [22]; and 3) staff available for remote technical support (Table 2: *Who provided*). Nevertheless, it seems that hybrid learners still faced technological problems which may have affected their learning experience. In future studies, more effort should be made in conducting and considering environmental conditions and students’ capabilities such as self-directedness when implementing Hybrid courses, as recommended by Atkins et al. [24]. Satisfaction surveys could be reviewed to include questions that compute most frequent problems to allow researchers to design specific strategies to mitigate them. Future studies should assess prospective students’ attitudes towards online learning, computer literacy, and skills to identify critical success factors the group of participants who could beneficiate from a blended learning approach.

Another possible explanation to the In-person larger effects could be related to participants habituation to in-person education [54]. Older students who are more familiar with traditional education systems that heavily rely on in-person training may still prefer that methodology and may find it more difficult to adapt to an unfamiliar learning environment [55]. This assumption could explain why participants ≥31 years old in the Hybrid group had the lowest increase in wheelchair service knowledge when compared with younger participants (Table 6). Moreover, it could suggest that the Hybrid learning approach is more effective in younger population.

Table 3 presents some training costs associated with this study’s training interventions, particularly, trainers’ stipend, staff support, food, and beverage during the training days. In these categories, the Hybrid courses had an overall 40% lower cost in India and 39% in Mexico when compared with the In-person courses. Although there are other costs related to facilitating these trainings that were not captured in this study, we believe that reducing the number of in-person training days decreases the cost associated with leading the training.

### Generalizability of results

Although randomized clinical trials are considered the gold standard for assessing the efficacy of an intervention and the generalizability of the results [56, 57], our goal was to design a pragmatic study that included the circumstances of practice that could hopefully transmit more relevant, actionable, and tailored results [58] in light of the recognition that evidence-based practice should be informed by practice-based evidence and research [58, 59]. As recommended by the literature, when non-randomized designs are being used to build evidence-based health practice, it is necessary to improve the quality of the reporting [57]. We used two widely-recognized tools to enhance the quality of the reporting of these interventions and, ultimately, their replicability: 1) The Transparent Reporting of Evaluations with Nonrandomized Designs (TREND) [57], used to describe the methodology; and 2) the Template for Intervention Description and Replication (TIDieR) (Table 2) [33], to provide a complete detailed report of all interventions and increase the potential impact of this study.

### Contribution to literature

To our knowledge, this trial is the only published study to date testing and comparing the effectiveness of a hybrid and in-person course in increasing basic level wheelchair service knowledge based on the WHO-WSTP. Previous studies have tested the Hybrid Course exclusively [22, 31], and we are unaware of any studies that have tested the in-person methodology suggested by the WHO WSTP-Basic Level. In the wheelchair service provision sector, the WHO WSTP-Basic in-person methodology is commonly used as the standard of practice for delivering training. This training approach was decided through a consensus-based process, and therefore does not have the benefit of evidence to demonstrate that is effective. Despite being considered the standard of practice, the in-person methodology showed only a small effect size superior to the hybrid methodology. This finding questions if the cost associated with leading an in-person training is justified by its impact when effective alternative learning methodologies are available. Our study suggests that both methodologies, a Hybrid and In-person course, are effective and well-accepted methods to train personnel in basic level wheelchair service provision.

### Limitations

Some limitations of this study are important to note in planning for future research and interpreting the current results. We did not randomize participants to the intervention groups. The lack of randomization cannot ensure that groups’ baseline characteristics will not differ. However, educational studies are difficult to mask learners to their assigned group, which may result in contaminating effects that further compromise the randomization process[60]. According to Sullivan, non-randomized methods are common in education research and considered by experts as not inferior to randomized clinical trials [60]. We strengthened the quality of our study by implementing the following factors used by Best Evidence Medical Education[60, 61]: 1) A “Pragmatic Trial” [62] that consisted of 2 interventions compared in real-world practice; 2) comparison group that received an active intervention; 3) training manuals or methods to ensure a detailed intervention; 4) multiple sites; 5) low dropout rates, and 6) a rigorous statistical method to confirm the findings, in this case, an intention-to-treat analysis based on multiple chain imputations and adjusted the models for potential confounders, interactions, and fixed and random effects.

The Satisfaction Surveys used in this study have not been validated in the target population. The Surveys were developed using previous satisfaction surveys and an international stakeholders’ group that guided the process and the Hybrid Satisfaction Survey has been previously used to measure levels of satisfaction among wheelchair providers [31]; nevertheless, until a formal validation process we are unsure if the tool measures the underlying outcome of interest [63].

We acknowledge that there were slight differences in the training interventions reported on the TIDieR table including trainers’ background, number of recitations, the language, and total wheelchair users who volunteer as role models (Table 2). The objective of this study was to compare the effectiveness of these interventions in real-world practice settings, with heterogenous populations, and flexibility to adjust the training to the context and resources available. Despite the diverse backgrounds of trainers, local master trainers with same qualifications across trainings coordinated the interventions. In terms of language, we combined groups to increase the sample size and to be able to generalize results regardless of whether it was delivered in English or Spanish. Nevertheless, we also analyzed the effectiveness of the training intervention by country and learning methodology; all groups had a statistically significant increase in total test scores after the training intervention (p <0.0001) (Table 5). Furthermore, Table 3 provided some information related to the trainings interventions’ costs. It is important to notice that we did not conduct a cost-effectiveness analysis between the Hybrid and the In-person courses. We encourage future studies to collect a complete dataset of costs and a rigorous analysis of the different training interventions.

Our sample size was estimated based on the WHO WSTP-B recommendations for the training groups’ size [34]. In addition, in planning for this study, we considered the budget of the project and decided that having multiple sites (India and Mexico) was more desirable than spending the allocated resources in one site with multiple trainings. After obtaining the data from the study, using the mean differences of the Hybrid and In-person groups (15.1 and 18.7), the pooled standard deviation (9.87), the number of clusters per intervention (2, India and Mexico), the size of each intervention (38 Hybrid and 43 In-person), and the ICC (7.244x10^-18^) we calculated the power of a clustered study for a two-tails test of means of 64%. If one side, the power will go up to 75%. Although our study did not reach the minimum power desirably, 80% [64], we consider our outcomes to be reliable based on the multiple imputations (11 times of main dataset) and the results of the sensitivity analysis (no changes in the significance after removing the imputations). It is important to note that we did not power the study based on the number of imputed participants; if so, the power of the study will be up to 90%. Most importantly, we are providing the data to estimate sample sizes for future studies and strengthen the quality of designs.

## Conclusion

Evidence highlights the global shortage of wheelchair service provision education and training that is related to inappropriate wheelchair provision. The results from this study suggest that both currently available learning methodologies, a hybrid and in-person course, are effective in increasing knowledge in basic level wheelchair service provision with overall high levels of satisfaction among participants.

To increase the number of training opportunities and to promote an equitable distribution in underserved areas, alternative learning methodologies need to be developed and tested in international settings. Blended learning is an attractive and sustainable learning approach that has demonstrated to be as effective as traditional educational strategies. In resourced constrained settings, where the need is great and resources are limited, lower-cost solutions that can significantly scale interventions and overcome knowledge dissemination barriers are critical to develop but should not compromise the learning quality. The lessons learned from this study could help organizations to develop strategies to mitigate potential implementation barriers and limitations and help to advance research in blended-learning training.

## Supporting information

S1 File ISWP In-person and Hybrid satisfaction surveys. (PDF)
