# Supplementary material for: Comparing the effectiveness of a hybrid and in-person courses of wheelchair service provision knowledge: A controlled quasi-experimental study in India and Mexico
Source: PLoS One. 2019 May 31;14(5):e0217872. doi: 10.1371/journal.pone.0217872 (PMC6544290; doi:10.1371/journal.pone.0217872)
Supplement: S1 File — (PDF) [file pone.0217872.s001.pdf]

# S1. Hybrid and In-person Satisfaction Survey English and Spanish

| Sub-domains | Hybrid Satisfaction Survey | In-person Satisfaction Survey |
|---|---|---|
| **Interaction** | E-learning sessions kept me alert and focused. | The course kept me alert and focused. |
| | The combination of e-learning sessions and in-person training kept me alert and focused. | |
| | I am satisfied with the quality of interaction between all involved parties (instructor and participants). | I am satisfied with the quality of interaction between all involved parties (instructor and participants). |
| | I am satisfied with the way I interacted with other students. | I am satisfied with the way I interacted with other students. |
| | I am satisfied with my participation in the class. | I am satisfied with my participation in the class. |
| **Instructor** | In the e-learning format, the instructor(s) was supportive and responsive to my questions. | The instructor(s) was supportive and responsive to my questions. |
| | In the in-person format, the instructor(s) was supportive and responsive to my questions. | |
| | I was satisfied with the accessibility and availability of the instructor(s). | I was satisfied with the accessibility and availability of the instructor(s). |
| | I continuously received feedback throughout this course. | I continuously received feedback throughout this course. |
| **Instruction methodology** | The use of e- learning technology in this course encouraged me to learn independently. | |
| | The use of hands-on activities in this course encouraged me to learn independently. | The use of hands-on activities in this course encourages me to learn independently. |
| | After this course, my understanding of wheelchair service provision has improved. | After this course, my understanding of wheelchair service provision has improved. |
| | I am satisfied with the level of effort this course required. | I am satisfied with the level of effort this course required. |
| | I am satisfied with my performance in this course. | I am satisfied with my performance in this course. |
| | I believe I will receive a passing score on the ISWP Basic Test after completing the training. | I believe I will receive a passing score on the ISWP Basic Test after completing the training. |
| | I am satisfied with how I will be able to apply what I have learned in this course. | I am satisfied with how I will be able to apply what I have learned in this course. |
| | I am willing to take another course that is part online and part in-person. | I am willing to take another course using in-person learning methodology. |
| | I enjoyed this course. | I enjoyed this course. |
| | I enjoyed working independently in the e-learning portion. | I enjoyed working on activities independently. |
| | I enjoyed working collaboratively in the in-person portion. | I enjoyed working collaboratively in the activities. |
| **Content** | The goals of this course were clearly stated at the beginning of the course. | The goals of this course were clearly stated at the beginning of the course. |
| | My expectations for this course were met. | My expectations for this course were met. |
| | In my opinion, the objectives of this course have been accomplished. | In my opinion, the objectives of this course have been accomplished. |
| | The WHO WSTP Reference Manual supported the learning outcomes for the course. | The WHO WSTP Reference Manual supported the learning outcomes for the course. |
| | Other reading materials assigned were relevant to the course objective. | Other reading materials assigned were relevant to the course objective. |
| | Overall, the content of the videos was relevant to the learning outcomes of the course. | Overall, the content of the videos was relevant to the learning outcomes of the course. |
| | Overall, the online activities were relevant to the learning outcomes of the course. | |
| | Overall, the in-person activities were relevant to the learning outcomes of the course. | Overall, the in-person activities were relevant to the learning outcomes of the course. |

| | | |
|---|---|---|
| | I am satisfied with this course and will recommend it to others. | I am satisfied with this course and will recommend it to others. |
| | The portion of time I spent doing online activities and time I spent in-person was appropriate. | |
| | The portion of time I spent doing online activities was appropriate. | |
| | The portion of time I spent in-person training was appropriate. | The time I spent in-person training was appropriate. |
| | The material that was selected for the online portion was appropriate. | |
| | The material that was selected for the in-person portion was appropriate. | |
| **Technology** | Course content shown or displayed on the screen was clear in the e-learning portion. | |
| | The video image was clear and comprehensive in the e-learning portion. | |
| | Overall, the e-learning ran smoothly with few technical difficulties. | |
| | Technical problems were not frequent in the e-learning portion. | |
| | Technical problems in the e-learning portion do not affect my understanding of the course. | |
| | The e-learning methodology is reliable. | |

At the end of each sub-domain the following open-ended question was included: *For the statements where you selected "strongly disagree" or "disagree" please provide suggestions to make your learning experience more satisfactory.*

# Encuesta de Satisfacción del Curso Semipresencial y Presencial
# Versión en español

| Subdominios | Encuesta de Satisfacción - Semipresencial | Encuesta de Satisfacción - Presencial |
|---|---|---|
| **Interacción** | Las sesiones en línea de este curso me mantuvieron alerto y concentrado. | Las sesiones de este curso me mantuvieron alerto y concentrado. |
| | La combinación de sesiones de aprendizaje en línea y sesiones presenciales me mantienen alerto y concentrado. | |
| | Estoy conforme con la calidad de la interacción entre los participantes e instructores de este curso. | Estoy conforme con la calidad de la interacción entre los participantes e instructores de este curso. |
| | Estoy satisfecho con la forma en la que interactué con otros participantes. | Estoy satisfecho con la forma en la que interactué con otros participantes. |
| | Estoy satisfecho con mi participación en este curso. | Estoy satisfecho con mi participación en este curso. |
| **Instructor** | En las sesiones en línea, los instructores respondieron a mis preguntas y me ofrecieron apoyo. | Durante el curso, los instructores respondieron a mis preguntas y me ofrecieron apoyo. |
| | En las sesiones presenciales, los instructores respondieron a mis preguntas y me ofrecieron apoyo. | |
| | Estoy conforme con la accesibilidad y disponibilidad de los instructores. | Estoy conforme con la accesibilidad y disponibilidad de los instructores. |
| | Recibí retroalimentación a lo largo del curso. | Recibí retroalimentación a lo largo del curso. |
| **Metodología de aprendizaje** | Los módulos en línea de este curso me motivaron a aprender de manera independiente. | |
| | Las sesiones presenciales de este curso me motivaron a aprender de manera independiente. | Las sesiones presenciales de este curso me motivaron a aprender de manera independiente. |
| | Después de este curso, mi comprensión del servicio de sillas de ruedas ha mejorado. | Después de este curso, mi comprensión del servicio de sillas de ruedas ha mejorado. |
| | Estoy conforme con el nivel de esfuerzo que este curso requirió. | Estoy conforme con el nivel de esfuerzo que este curso requirió. |
| | Estoy conforme con mi desempeño en este curso. | Estoy conforme con mi desempeño en este curso. |
| | Creo que aprobaré el Examen Básico de ISWP una vez finalizado el curso. | Creo que aprobaré el Examen Básico de ISWP una vez finalizado el curso. |
| | Estoy conforme con cómo aplicaré los conocimientos obtenidos en este curso. | Estoy conforme con cómo aplicaré los conocimientos obtenidos en este curso. |
| | Estoy dispuesto(a) a tomar otro curso semipresencial. | Estoy dispuesto(a) a tomar otro curso de metodología presencial. |
| | Me gustó este curso. | Me gustó este curso. |
| | Disfruté trabajar de forma independiente en la porción en línea. | Disfruté trabajar de forma independiente. |
| | Disfruté trabajar de forma colaborativa en la porción presencial. | Disfruté trabajar de forma colaborativa en actividades. |
| **Contenido** | Los objetivos de este curso se especificaron con claridad al comienzo del curso. | Los objetivos de este curso se especificaron con claridad al comienzo del curso. |
| | El curso cumplió mis expectativas. | El curso cumplió mis expectativas. |
| | En mi opinión, se cumplieron los objetivos de este curso. | En mi opinión, se cumplieron los objetivos de este curso. |
| | El manual de referencia de la OMS apoyó el cumplimiento de los objetivos del curso. | El manual de referencia de la OMS apoyó el cumplimiento de los objetivos del curso. |
| | Otros materiales de lectura asignados fueron relevantes para el cumplimiento de los objetivos del curso. | Otros materiales de lectura asignados fueron relevantes para el cumplimiento de los objetivos del curso. |
| | En general, el contenido de los videos fue relevante para el cumplimiento de los objetivos del curso. | En general, el contenido de los videos fue relevante para el cumplimiento de los objetivos del curso. |
| | En general, las actividades en línea fueron relevantes para el cumplimiento de los objetivos del curso. | |

| | | |
|---|---|---|
| | En general, las actividades presenciales fueron relevantes para el cumplimiento de los objetivos del curso. | En general, las actividades presenciales fueron relevantes para el cumplimiento de los objetivos del curso. |
| | Estoy satisfecho con este curso y se lo recomendaría a otros. | Estoy satisfecho con este curso y se lo recomendaría a otros. |
| | La cantidad de tiempo que invertí haciendo actividades en línea y la cantidad de tiempo que invertí en actividades presenciales fue adecuada. | |
| | La cantidad de tiempo que invertí haciendo actividades en línea fue adecuada. | |
| | La cantidad de tiempo que invertí realizando actividades presenciales fue adecuada. | La cantidad de tiempo que invertí realizando actividades presenciales fue adecuada. |
| | El material que se seleccionó para la parte en línea fue apropiado. | |
| | El material que se seleccionó para la parte presencial fue apropiado. | |
| **Tecnología** | El contenido del curso mostrado en pantalla durante la porción en línea, fue claro. | |
| | La imagen de video, durante la porción en línea, fue clara. | |
| | En general, mi aprendizaje en línea transcurrió sin problemas, sólo existieron algunas dificultades técnicas. | |
| | Los problemas técnicos no fueron frecuentes en la porción en línea. | |
| | Los problemas técnicos en la porción en línea no afectaron mi comprensión del curso. | |
| | La metodología de aprendizaje en línea es confiable. | |

Al final de cada subdominio se incluyó la siguiente pregunta abierta: *En los enunciados en los que seleccionó "totalmente en desacuerdo" o "desacuerdo", por favor proporcione sugerencias que nos permitan mejorar su experiencia de aprendizaje.*
